# Lipid Metabolism and Signaling in Tumors and Cerebrovascular Diseases

**DOI:** 10.3390/ijms23116280

**Published:** 2022-06-03

**Authors:** Laura Gatti

**Affiliations:** Laboratory of Neurobiology and UCV, Neurology IX Unit, Fondazione IRCCS Istituto Neurologico Carlo Besta, 20133 Milan, Italy; laura.gatti@istituto-besta.it

The aim of this Special Issue was to update readers regarding state-of-the-art research into lipid metabolism and signaling in tumors and cerebrovascular diseases.

Lipid molecules have been reported to regulate a broad range of cellular functions, from cell signaling and membrane dynamics to intercellular communication and gene expression. Due to the lipid heterogeneity, it has been estimated that the human lipidome may be composed by 100,000 different lipid species [1]. Lipids play multiple roles in brain function, affecting the elasticity and structural organization of synaptic membranes and modulating protein activity involved in cellular signaling. Therefore, also cerebrovascular diseases that considerably threaten brain health are likely to be associated with lipid deregulation. A large body of evidence supports that lipid metabolism is implicated in the development of cancer by promoting cell proliferation and survival and aggressive behavior [2], with changes in lipid metabolism favoring cell migration and invasion [3]. In tumor cells, the stimulation of lipid synthesis may result from the activation of oncogenic pathways, with newly synthesized lipids preferentially becoming phospholipids and participating in cell signaling.

Thus, knowledge regarding lipid homeostasis represents a powerful tool providing another layer of details at the molecular and cellular levels, which may help in the exploration of novel biomarkers and new options for treatment of diseases affecting a large number of patients. The Special Issue was conceived as an editorial project to bring together basic researchers and clinicians working in the area of lipid metabolism, in humans and cellular models, to gain insights into the role of lipids involved in pathophysiology of tumors and cerebrovascular diseases and provide an overview of the most recent advances.

In the present Special Issue, seven cutting edge scientific papers in the field—four original articles and three literature review papers—are introduced, highlighting recent advances both in cancer and in cerebrovascular research. Notably, three out of seven papers are focused on tumors of central (glioblastoma, GBM; anaplastic ganglioglioma, AGGL) or peripheral (peripheral nerve sheat tumors, PNSTs) nervous system.

Di Ianni N. and colleagues discussed the unique or alternative source of energy exploited by GBM cells, exploring how deprivation of specific nutrients and accumulation of toxic byproducts can induce T-cell dysfunction [4]. Lipid metabolism reprogramming in GBM is still poorly described and can include alterations in fatty acids (FA) transport, storage in lipid droplets and oxidation to generate ATP. GBM develops in a lipid-rich environment but the environmental lipids can be relatively unavailable, as they are generally already engaged in normal neural elements, including myelin. GBM cells need large amounts of lipids for many structural and energetic functions. The activity of de novo lipid generation is crucial for the generation of all the components necessary for the membrane constitution, and for GBM growth and survival. Understanding the metabolic programs of these cellular components and how they impact fitness or dysfunction will be useful to improve treatment modalities, including immunotherapeutic strategies, against the most aggressive and lethal primary brain tumor. Some critical dietary modifications, that affect nutrient availability, are exploited in clinical studies to slow tumor progression and improve antitumor immunity. There is an important correlation between hypocaloric or ketogenic diets and immune response. These conditions can be taken into consideration to enhance the efficacy of some immunotherapeutic modalities.

An innovative approach combining mass spectrometry and thin-layer chromatography has been performed by Fabris D. et al. to provide a comparative characterization of ganglioside composition in a neuronal–glial type of tumor (AGGL), vs. peritumoral (PT) and normal brain tissues (NB). AGGL ganglioside composition was found to be highly altered as compared to peritumoral PT and NB tissues, including changes in both carbohydrate and ceramide residues of ganglioside species [5]. The additional contribution of this study is the characterization of the ganglioside composition in valuable and rarely accessible peritumoral tissues, since the important biochemical changes that occur within this area are considered responsible for tumor cell infiltration into the surrounding NB, promoting tumor recurrence and drug resistance.

Considering that lipid homeostasis plays a crucial role also in fibrotic/inflammatory processes in several cancers, Vetrano I.G. and colleagues have investigated the specific factors contributing to the more aggressive behavior of some PNSTs which are benign tumors, according to their biological behavior, but have the potential for malignant degeneration [6]. They include schwannomas, neurofibromas (NFs), and plexiform neurofibromas (PNFs), among others. The untargeted and targeted lipidomic approaches performed in the study contributed to better clarify the role of bioactive lipids in PNST natural history and to highlight disease molecular features and pathogenesis. Such findings, by suggesting that different subtypes of PNSTs present peculiar lipid profiles, will help to identify possible targets for precision medicine in these rare tumors.

In their study, Lai M. and co-authors examined the role of acid ceramidase in maintaining cellular homeostasis, through the regulation of autophagy and mitochondrial activity in melanoma cell lines [7]. In fact, sphingolipid signaling plays a pivotal role in melanoma progression and metastasis through overexpression of lysosomal acid ceramidase (AC), which catalyzes the hydrolysis of pro-apoptotic long chain ceramides into sphingosine and fatty acid. The present work sheds light on a mechanism whereby AC expression hinders metabolic equilibrium of melanoma cells by means of autophagy impairment and mitochondrial dysfunction. The reported findings suggest that after AC-ablation, melanoma cells change and adapt their metabolism to survive in the absence of AC, although in a way that does not allow them to cope with the stress of nutrient deprivation. These results may be ideally exploited to develop AC inhibitors as adjuvant molecules for chemo- and radio-therapy in melanoma.

To stay on the topic of cancer treatment, Giussani P. et al. in their review article discussed how sphingolipids are involved in interactions between tumor cells and the immune system and how knowledge in this topic could be useful to enhance the efficacy of different immunotherapy approaches [8]. In particular, they explored how sphingolipids are pivotal components of plasma membranes and could modulate the functionality of surface receptors expressed also by immune cells. In this context, they investigated the role of bioactive mediators, such as sphingosine 1-phosphate and ceramide, that could significantly affect lymphocyte functions in the tumor microenvironment, and they elucidated how it is possible to employ sphingolipids as antigen targets.

The role of thromboxane in ischemic stroke has been addressed by Szczuko M. and colleagues in their review article, that is focused also on effective therapies for the treatment of this important disorder [9]. The effectiveness of acetylsalicylic acid (ASA) in the prevention of ischemic stroke can be defined by determining its biologically active substance, which is thromboxane. In fact, ASA blocks cyclooxygenase and thromboxane synthesis, thus the inclusion of ASA in the prevention of stroke has a beneficial effect that is associated with the effect on thromboxane. The studies examined here showed that an initial high concentration of thromboxane B2 (TXB2) may be a risk factor for ischemic stroke or ischemic heart disease. However, there is insufficient evidence to suggest that TXB2 could be used in clinical practice as a marker of ischemic stroke.

Another cerebrovascular disease was investigated by Dei Cas M. et al., in an original research article concerning Moyamoya arteriopathy (MA), a rare and poorly known condition characterized by both ischemic and hemorrhagic strokes [10]. Since lipids are implicated in modulating neo-vascularization, angiogenesis, and inflammation, that are typical hallmarks of MA, their deregulation is potentially involved in the disorder. By mass spectrometry, the authors have performed a complete plasma lipidomic analysis of MA patients and control subjects, aimed at unraveling the complexity of MA pathogenesis. Their findings indicated that lipid signature could play a central role in MA and evidenced that the plasma lipid profile of MA patients is definitely peculiar, thus highlighting a novel source of reliable clinically useful circulating biomarkers for such rare disease.

We are confident that scientists, including all the authors involved in this Special Issue, will continue to provide unexpected discoveries that will lead us to understand the role of lipids in cellular metabolism and signaling of tumors and cerebrovascular diseases This, in turn, will encourage us to develop novel promising preventive and therapeutic strategies for patients.
